# Positive logics

**DOI:** 10.1007/s00153-022-00837-3

**Published:** 2022-07-09

**Authors:** Saharon Shelah, Jouko Väänänen

**Affiliations:** 1 Institute of Mathematics, Hebrew University, Jerusalem, Israel; 2 Rutgers University, New Jersey, USA; 3 Department of Mathematics and Statistics, University of Helsinki, Helsinki, Finland; 4 ILLC, University of Amsterdam, Amsterdam, Netherlands

**Keywords:** Lindström’s Theorem, Abstract model theory, Existential second order, 03C95, 03C85, 03C68

## Abstract

Lindström’s Theorem characterizes first order logic as the maximal logic satisfying the Compactness Theorem and the Downward Löwenheim-Skolem Theorem. If we do not assume that logics are closed under negation, there is an obvious extension of first order logic with the two model theoretic properties mentioned, namely existential second order logic. We show that existential second order logic has a whole family of proper extensions satisfying the Compactness Theorem and the Downward Löwenheim-Skolem Theorem. Furthermore, we show that in the context of negation-less logics, *positive logics*, as we call them, there is no strongest extension of first order logic with the Compactness Theorem and the Downward Löwenheim-Skolem Theorem.

## Introduction

Our motivating question in this paper is whether we can generalize Lindström’s Theorem from first order logic to $\Sigma ^1_1$, that is, existential second order logic. In the case of first order logic Lindström’s Theorem says that first order logic is maximal with the Compactness Theorem^1^ and the Downward Löwenheim-Skolem Theorem^2^ among logics satisfying some minimal closure conditions [10]. One of the assumed closure conditions is closure under negation. What happens if we drop this assumption? It seems that this question was first explicitly raised in [7]. The Compactness Theorem and the Downward Löwenheim-Skolem Theorem make perfect sense, whether we have negation or not. These two conditions make no reference to negation.

In earlier related work ([14]) we showed that a strong form of Lindström’s Theorem fails for extensions of $L_{\kappa \omega }$ and $L_{\kappa \kappa }$: For weakly compact $\kappa$ there is no strongest extension of $L_{\kappa \omega }$ with the $(\kappa ,\kappa )$-compactness property and the Löwenheim-Skolem Theorem down to $\kappa$. With an additional set-theoretic assumption, there is no strongest extension of $L_{\kappa \kappa }$ with the $(\kappa ,\kappa )$-Compactness Theorem and the Löwenheim-Skolem theorem down to $<\kappa$.

Obviously first order logic itself is not maximal if negation is dropped because existential second order logic $\Sigma ^1_1$, and even $\Sigma ^1_{1,\delta }$ (also denoted $PC_\Delta$), i.e. existential second order quantifiers followed by a countable conjunction of first order sentences, which clearly satisfy both the Compactness Theorem and the Downward Löwenheim-Skolem Theorem, also properly extend first order logic.

We are led to the following (interrelated) questions, all in the context of logics where closure under negation is *not* assumed:

### Question 1

Is $\Sigma ^1_1$ (or rather $\Sigma ^1_{1,\delta }$) maximal among logics satisfying the Compactness Theorem and the Downward Löwenheim-Skolem Theorem?

### Question 2

Is there an extension of $\Sigma ^1_1$ (or $\Sigma ^1_{1,\delta }$) which is maximal among logics satisfying the Compactness Theorem and the Downward Löwenheim-Skolem Theorem?

### Question 3

Is there a characterization of $\Sigma ^1_1$ (or $\Sigma ^1_{1,\delta }$) as maximal among logics satisfying some model-theoretic conditions?

### Question 4

Is there an extension of $\Sigma ^1_1$ (or $\Sigma ^1_{1,\delta }$) which is maximal (or even strongest) among logics satisfying some model-theoretic conditions?

In this paper we formulate Questions 1 and 2 in exact terms. We answer Question 1 negatively. As to Question 2 we show that there is no strongest^3^ extension of $\Sigma ^1_1$ satisfying the Compactness Theorem and the Downward Löwenheim-Skolem Theorem. The existence of a maximal one (which has no proper such extension) remains open. Questions 3 and 4 remain completely unanswered. Admittedly, Question 4 is a little vague as both “extension” and “model-theoretical conditions” are left open.

To answer the above Questions 1 and 2 we introduce a family of new generalized quantifiers associated with the very natural and intuitive concept of the density of a set of reals. These quantifiers are defined for the purpose of solving the said questions and may lack wider relevance, although the general study of logics without negation is so undeveloped that it may be too early to say what is relevant and what is not.

**Notation:** We use $\mathcal {M}$ and $\mathcal {N}$ to denote structures, and *M* and *N* to denote their universes, respectively. For finite sequences *s* and sets *a*, we use  to denote the extension of *s* by the set *a*. For sequences of length $\le \omega$, $s\triangleleft s'$ means that *s* is an initial segment of $s'$. The empty sequence is denoted $\emptyset$. A subset *A* of $2^\omega$ is said to be *dense* if for all $s\in 2^{<\omega }$ there is $s'\in A$ such that $s\triangleleft s'$. We use $\mathbb {P}(\omega )$ to denote the power-set of $\omega$.

## Positive logics

We define the concept of a *positive logic,* meaning a logic without negation, except in front of atomic (and first order) formulas. We have to be careful about substitution in this context. If we are too lax about the substitution^4^ of formulas into atomic formulas we end up having a logic which is closed under negation, which is not what we want. Substitution is very natural, but it is not needed in Lindström’s characterization of first order logic.

One may ask whether a logic deserves to be called a *logic* if it is not closed under negation? We do not try to answer this question, but merely point out that there are several logics that do not have a negation in the sense that we have in mind, i.e. in the sense of classical logic. Take, for example, constructive logic. Although it has a negation, it does not have the Law of Excluded Middle, so its negation does not function in the way we mean when we ask whether a logic is closed under negation. In our sense constructive logic is not closed under negation. Another example is continuous logic [3] and the related positive logic of [4]. We have already mentioned existential second order logic $\Sigma ^1_1$ and its stronger form, $\Sigma ^1_{1,\delta }$. In the same category as $\Sigma ^1_1$ are Dependence logic [15] and Independence Friendly Logic [11]. Transfinite game quantifiers yield infinitary logics which are not closed under negation, due to non-determinacy [8]. In the finite context there is the complexity class non-deterministic polynomial time NP, which is equivalent to existential second order logic on finite models, of which it is not known whether it is closed under negation. In this paper we introduce new examples of logics without negation.

### Definition 1

A *positive logic* is an abstract logic^5^ in the sense of [10] (see also [6]) which contains first order logic and is closed under disjunction, conjunction, and first order quantifiers $\exists$ and $\forall$. We do not require closure under negation, nor closure under substitution.

### Example 2

1. First order logic is a positive logic.
2. $\Sigma ^1_1$ and $\Sigma ^1_{1,\delta }$ are positive logics.
3. If *L* is a positive logic, then so is $\Sigma ^1_1(L)$, the closure of *L* under existential second order quantification.

## A class of new quantifiers

In the tradition of [9] we define our new generalized quantifiers by first specifying a class of structures, closed under isomorphisms.

Let $\tau _d$ be the vocabulary $\{R_0,R_1,R_2,R_3,R_4\}$ consisting of binary predicates $R_0,R_1,R_2$ and unary predicates $R_3,R_4$.

### Example 3

A canonical example of a $\tau _d$-structure is the model

$$\begin{aligned} \mathcal {M}_{A}=(M,R^{\mathcal {M}_A}_0,R^{\mathcal {M}_A}_1,R^{\mathcal {M}_A}_2,R^{\mathcal {M}_A}_3, R^{\mathcal {M}_A}_4), \end{aligned}$$

where $$A\subseteq 2^\omega $$ and

- $$M=2^{<\omega }\cup A$$.
($$i=0,1$$).
- $$R_2^{\mathcal {M}_A}=\{(a,b)\in M\times M: a\triangleleft b\}$$.
- $$R_3^{\mathcal {M}_A}=\{\emptyset \}.$$
- $$R_4^{\mathcal {M}_A}=M.$$

### Definition 4

For $n<\omega$ and $\eta \in 2^n$ we define $\psi _\eta (x)$ as:

$$\begin{aligned} \begin{array}{rl} R_4(x)\wedge \exists y_0\ldots \exists y_n&(R_3(y_0)\wedge \bigwedge _{i\le n}R_4(y_i)\wedge \bigwedge _{i<n} y_iR_{\eta (i)}y_{i+1}\wedge \bigwedge _{i\le n}y_i R_2 x). \end{array} \end{aligned}$$

For a $\tau _d$-model ${\mathcal {M}}$ and $a\in M$ we define

$$\begin{aligned} \begin{array}{l} \Omega ({{\mathcal {M}}, a})=\{\eta \in 2^{<\omega }: {\mathcal {M}}\models \psi _\eta (a)\}\\ \Omega ({\mathcal {M}})=\{\eta \in 2^{\omega } : \text{ for } \text{ some }\, a\in M, \eta \restriction n\in \Omega ({{\mathcal {M}},a}) \text{ for } \text{ all }\, n<\omega \}. \end{array} \end{aligned}$$

If $\eta \in \Omega ({{\mathcal {M}}, a})$, we say that *a* *represents* $\eta$ in ${\mathcal {M}}$. We also say that ${\mathcal {M}}$ *represents* the set $\Omega ({\mathcal {M}})$.

One element *a* can represent several $\eta$, but later in Sect. 7 we impose a further restriction to the effect that representation is unique.

Note that if ${\mathcal {M}}$ is a $\tau _d$-model, then the property “$\Omega ({\mathcal {M}})$ is dense” is a $\Sigma ^1_1$-property of ${\mathcal {M}}$. Since we aim at a logic which goes beyond existential second order logic, we have to sharpen the requirement of density. The property of $\tau _d$-models we are interested in is the property that “$\Omega ({\mathcal {M}})\setminus A$ is dense” for some preassigned set $A\subseteq 2^\omega$ of reals.

### Definition 5

Let $A\subseteq 2^\omega$. We define the Lindström quantifier $Q_A$ as follows. Suppose $\mathcal {M}$ is a model and $\bar{c}\in M^k$. Then we define

1 $$\begin{aligned} (Q_Ax_0x_1)(\psi _0(x_0,x_1,\bar{c}),\psi _1(x_0,x_1,\bar{c}),\psi _2(x_0,x_1,\bar{c}),\psi _3(x_0,\bar{c}),\psi _4(x_0,\bar{c})) \end{aligned}$$

to be true in $\mathcal {M}$ if and only if $\Omega (\mathcal {M}_{\bar{\psi }})\setminus A$ is dense, where

$$\begin{aligned} \bar{\psi }= & {} (\psi _0(x_0,x_1,\bar{c}),\psi _1(x_0,x_1,\bar{c}),\psi _2(x_0,x_1,\bar{c}),\psi _3(x_0,\bar{c}),\psi _4(x_0,\bar{c})),\\ \mathcal {M}_{\bar{\psi }}= & {} (M,R^\mathcal {N}_0,R^\mathcal {N}_1,R^\mathcal {N}_2,R^\mathcal {N}_3, R^\mathcal {N}_4),\end{aligned}$$

and

- $$R_i^\mathcal {N}=\{(a,b)\in M^2: {\mathcal {M}}\models \psi _i(a,b,\bar{c})\}$$ ($$i=0,1,2$$).
- $$R_3^\mathcal {N}=\{a\in M:{\mathcal {M}}\models \psi _3(a,\bar{c})\}$$.
- $$R_4^\mathcal {N}=\{a\in M : {\mathcal {M}}\models \psi _4(a,\bar{c})\}$$.

### Definition 6

Suppose $A\subseteq 2^\omega$. We define the positive logic $L^d_A$ as the closure of first order logic under conjunction, disjunction, first order quantifiers $\exists$ and $\forall$, the existential second order quantifier $\exists R$, where *R* is a relation symbol, and the generalized quantifier $Q_A$. We denote by $L^{d,\omega }_A$ the extension of $L^d_A$ obtained by allowing countable conjunctions as a logical operation. Finally, the proper class $L^{d,\infty }_A$ denotes the extension of $L^d_A$ obtained by allowing arbitrary set-size conjunctions as a logical operation.

With the obvious definition of what it means for a positive logic to be a sublogic of another, we can immediately observe that $\Sigma ^1_1$ is a sublogic of $L^d_A$, and $\Sigma ^1_{1,\delta }$ is a sublogic of $L^{d,\omega }_A$ whatever *A* is.

### Example 7

Suppose $A\subseteq 2^\omega$. The class $K_A$ of $\tau _d$-models $\mathcal {M}$ satisfying “$\Omega (\mathcal {M})\setminus A$ is dense” is (trivially) definable in $L^d_A$, as $\mathcal {M}\in K_A$ if and only if $\mathcal {M}\models \psi _A$, where

$$\begin{aligned} \psi _A=(Q_Ax_0x_1)(R_0(x_0,x_1),R_1(x_0,x_1),R_2(x_0,x_1),R_3(x_0),R_4(x_0)).\end{aligned}$$

The model $\mathcal {M}_B$ of Example 3 is in $K_A$, if and only if $B\setminus A$ is dense.

For future reference we make the following observation: If $\eta \in 2^n$, $\bar{y}=(y_0,\ldots ,y_{n-1})$, $\bar{\psi }$ as in Definition 5 is a 5-tuple of formulas of $L^d_A$, $\bar{z}=(z_0,\ldots ,z_{k-1})$, and $\Gamma ^{n,k}_{\bar{\psi },\eta }(\bar{y},x,\bar{z})\in L^d_A$ is the conjunction of

$$\begin{aligned} \begin{array}{ll} \psi _4(y_i,\bar{z})&{} \text{ for }\, {i\le n}\\ \psi _4(x,\bar{z})\wedge \psi _3(y_0,\bar{z})\\ \psi _{\eta (i)}(y_i,y_{i+1},\bar{z})&{} \text{ for }\, {i< n}\\ \psi _2(y_i,x,\bar{z})&{} \text{ for }\, {i\le n},\\ \end{array} \end{aligned}$$

then (1) is equivalent to

2 $$\begin{aligned} \begin{array}{l} \text{ For } \text{ every }\, \sigma \in 2^{<\omega }\, \text{ there } \text{ are }\, \eta \in 2^\omega \setminus A\, \text{ extending } \,\sigma \\ \text{ and }\, a\in M\, \text{ such } \text{ that } \text{ for } \text{ some } \text{ function }\, n\mapsto \langle b^n_0,\ldots ,b^n_{n-1}\rangle \\ \text{ from }\, \omega \, \text{ to }\, M^n\, \text{ we } \text{ have } \mathcal {M}\models \Gamma ^{n,k}_{\bar{\psi },\eta \restriction n}(b^n_0,\ldots ,b^n_{n-1},a,\bar{c}) \text{ for } \text{ all }\, n<\omega . \end{array} \end{aligned}$$

We proceed to proving that the logic $L^d_A$, for suitably chosen $A\subseteq 2^\omega$, satisfies the Compactness Theorem and the Downward Löwenheim-Skolem Theorem, and also properly extends $\Sigma ^1_{1}$.

## The Compactness Theorem

We use the well-established method of ultraproducts to prove the Compactness Theorem of $L^d_A$.

### Theorem 8

(Łoś Lemma for $$L^d_A$$) Suppose $$2^\omega \setminus A$$ is dense. Suppose $${\mathcal {M}}_i$$, $$i\in I$$, are models and *D* is an ultrafilter on a set *I*. Let $${\mathcal {M}}=\prod _{i\in I}{\mathcal {M}}_i/D$$, $$f_0,\ldots ,f_{n-1}\in \prod _{i\in I}M_i$$ and $$\phi (x_0,\ldots ,x_{n-1})$$ in $$ L^d_A$$ (or even in $$L^{d,\infty }_A$$). Then

$$\begin{aligned} \{i\in I : {\mathcal {M}}_i\models \phi (f_0(i),\ldots ,f_{n-1}(i))\}\in D\Rightarrow {\mathcal {M}}\models \phi (f_0/D,\ldots ,f_{n-1}/D).\end{aligned}$$

### Proof

We use induction on $\phi$. The cases corresponding to the atomic formulas, the negated atomic fromulas, conjunction (even infinite conjunction), disjunction, $\exists$, $\forall$, and $\exists R$ (see e.g. [5, 4.1.14]) are all standard and well known. In the case of disjunction we use the property of ultrafilters that $I_1\cup I_2\in D$ implies $I_1\in D$ or $I_2\in D$. We are left with the induction step for $Q_A$. Let us denote $f_0(i),\ldots ,f_{n-1}(i)$ by $\bar{f}(i)$ and $f_0/D,\ldots ,f_{n-1}/D$ by $\bar{f}/D$. We assume

3 $$\begin{aligned} J=\{u\in I : \mathcal {M}_i\models (Q_Ax_0x_1)(\psi _0(x_0,x_1,\bar{f}(i)), \ldots ,\psi _4(x_0,\bar{f}(i)))\}\in D \end{aligned}$$

and demonstrate $\mathcal {M}\models (Q_A x_0x_1)(\psi _0(x_0,x_1,\bar{f}/D), \ldots ,\psi _4(x_0,\bar{f}/D))$. For $i\in J$ the set $B_i$ of elements $\eta$ of $2^\omega \setminus A$ such that there are $a_i\in M_i$ and $b^n_{0,i}\ldots ,b^n_{n-1,i}$ in $M_i$ such that $\mathcal {M}_i\models \Gamma ^{n,k}_{\bar{\psi },\eta \restriction n}(b^n_{0,i},\ldots ,b^n_{n-1,i},a_i,\bar{f}(i))$ for all $n<\omega$, is dense.

**Case 1:** *D* **is** $\aleph _1$**-incomplete.** Let $J=I_0\supseteq I_1\supseteq \ldots$ be a descending chain in *D* with empty intersection. We show that the set *B* of $\eta \in 2^\omega$ such that there is $a\in M$ such that for some $b^n_0,\ldots ,b^n_{n-1}$ in $\prod _iM_i/D$ we have $\mathcal {M}\models \Gamma ^{n,k}_{\bar{\psi },\eta \restriction n}(b^n_0,\ldots ,b^n_{n-1},a,\bar{f}/D)$ for all $n<\omega$, is the full set $2^\omega$. Since we assume that $2^\omega \setminus A$ is dense, it follows that $B\setminus A$ is dense, as we claim.

Suppose $\eta \in 2^\omega$ is arbitrary. Let $i\in I_{n+1}\setminus I_n$. Because $B_i$ is dense, there are, for all $n<\omega$, extensions $\eta _i\in 2^\omega$ of $\eta \restriction n$ and elements $a_i,b^n_{i,0},\ldots ,b^n_{i,n-1}\in M_i$ such that

4 $$\begin{aligned} \mathcal {M}_i\models \Gamma ^{n,k}_{\bar{\psi },\eta _i\restriction n}(b^n_{i,0},\ldots ,b^n_{i,n-1},a_i,\bar{f}(i)) \end{aligned}$$

for all $n<\omega$. Let $h(i)=a_i$. For $i\in I_n\setminus I_{n+1}$ and $m<n$, let $g^n_m(i)=b^n_{i,m}$. Now

$$\begin{aligned} \{i\in I : \mathcal {M}_i\models \Gamma ^{n,k}_{\bar{\psi },\eta \restriction n}(g^n_0(i),\ldots ,g^n_{n-1}(i),h(i),\bar{f}(i))\}\supseteq I_{n+1}\in D.\end{aligned}$$

Hence

$$\begin{aligned} \mathcal {M}\models \Gamma ^{n,k}_{\bar{\psi },\eta \restriction n}(g^n_0/D,\ldots ,g^n_{n-1}/D,h/D,\bar{f}/D).\end{aligned}$$

**Case 2:** *D* **is** $\aleph _1$ **-complete.** We show that the set *B* of $\eta \in 2^\omega \setminus A$ such that for some $a, b^n_0,\ldots ,b^n_{n-1}\in M$ we have $\mathcal {M}\models \Gamma ^{n,k}_{\bar{\psi },\eta \restriction n}(b^n_0,\ldots ,b^n_{n-1},a,\bar{f}/D)$ for all $n<\omega$, is dense. Suppose $\eta \in 2^{n}$. By the density of $B_i$, for each $i\in J$ there is $\nu _i\in B_i$ extending $\eta$. There is $J_0\subseteq J$ in *D* such that $\nu _i(n)$ is constant for $i\in J_0$. There is $J_1\subseteq J_0$ in *D* such that $\nu _i(n+1)$ is constant for $i\in J_{1}$, etc. By $\aleph _1$-completeness we get $J_\infty \in D$ such that $\nu _i(m)$ is constant, say $\eta ^*(m)$ for all $m\ge n$ and all $i\in J_\infty$. Now $\eta ^*\in B$ follows easily. $\square$

### Corollary 9

If $2^\omega \setminus A$ is dense, then:

1. $L^d_A$ (even $L^{d,\infty }_A$) satisfies the (full) Compactness Theorem.
2. Every sentence of $L^d_A$ with an infinite model has arbitrarily large models.
3. The only sentences in $L^d_A$ that have a negation (in the usual sense) are the first order (equivalent) ones.

### Proof

The usual argument gives 1: Suppose *T* is a finitely consistent theory in $L^d_A$. Let *I* be the set of finite subsets of *T* and for each $i\in I$, let $\mathcal {M}_i\models i$. If $\phi \in T$, let $A_\phi =\{i\in I : \phi \in i\}$. Then the family $\mathcal{J}=\{A_\phi : \phi \in T\}$ has the finite intersection property. Let *D* be a non-principal ultrafilter on *I* extending $\mathcal {J}$. Now if $\phi \in T$, then $\prod _D\mathcal {M}_i\models \phi$, as $\{i\in I : \mathcal {M}_i\models \phi \}\supseteq A_\phi \in D.$

Claim 2 follows immediately from Claim from 1. Claim 3 follows from the ultraproduct characterization of first order model classes (see e.g. [5,  4.1.12]) and the characterization of elementary equivalence in terms of ultrapowers [13]. $\square$

### Theorem 10

(Robinson’s Consistency Lemma for $L^d_A$) Suppose $2^\omega \setminus A$ is dense. Suppose $T_1$ and $T_2$ are consistent $L^d_A$-theories with vocabularies $\tau _1$ and $\tau _2$, respectively, such that $T_1\cap T_2$ is complete with respect to first order logic in the vocabulary $\tau _1\cap \tau _2$. Then $T_1\cup T_2$ is consistent.

### Proof

This proof is not specific to $L^d_A$, but is rather a well-known consequence of Łoś Lemma, Theorem 8. Let ${\mathcal {M}}_1\models T_1$ and ${\mathcal {M}}_2\models T_2$. Let ${\mathcal {M}}_l^-$ be the reduct of ${\mathcal {M}}_l$ to the vocabulary $\tau _d=\tau _1\cap \tau _2$. Now ${\mathcal {M}}_1^-$ and ${\mathcal {M}}_2^-$ are elementarily equivalent in first order logic, for if ${\mathcal {M}}_1^-\models \phi$ then necessarily $T_1\cap T_2\models \phi$, whence ${\mathcal {M}}_2^-\models \phi$, and vice versa. By [13] there are a set *I* and an ultrafilter *D* on *I* such that if we denote ${\mathcal {M}}_1^I/D$ by ${\mathcal {N}}_1$ and ${\mathcal {M}}_2^I/D$ by ${\mathcal {N}}_2$, then ${\mathcal {N}}_1\restriction \tau _d\cong {\mathcal {N}}_2\restriction \tau _d$. W.l.o.g. ${\mathcal {N}}_1\restriction \tau _d= {\mathcal {N}}_2\restriction \tau _d$. Let ${\mathcal {N}}$ be a common expansion of ${\mathcal {N}}_1$ and ${\mathcal {N}}_2$. By Theorem 8, ${\mathcal {N}}\restriction \tau _1\models T_1$ and ${\mathcal {N}}\restriction \tau _2\models T_2$. Hence, ${\mathcal {N}}\models T_1\cup T_2$. $\square$

## The Downward Löwenheim-Skolem property

The Downward Löwenheim-Skolem Property, which says that any sentence (of the logic) which has a model has a countable model, is an important ingredient of the Lindström characterization of first order logic. The main examples of logics with this property, apart from first order logic, are $L_{\omega _1\omega }$ and its sublogics $L(Q_0)$ (with the quantifier “there exists infinitely many”, see e.g. [1,  p. 8]) and the weak second order logic $L^2_w$ (with quantifiers for variables that range over finite sets, see e.g. [1,  p. 9]). We now prove this property for $L^d_A$ in a particularly strong form.

Because of lack of negation the elementary submodel relation $\mathcal {M}\preccurlyeq \mathcal {N}$ splits into two different concepts $\mathcal {M}\preccurlyeq ^+\mathcal {N}$ and $\mathcal {M}\preccurlyeq ^-\mathcal {N}$:

### Definition 11

Let us write $\mathcal {N}\preccurlyeq ^-_{L^d_A}\mathcal {M}$ if $\mathcal {N}\subseteq \mathcal {M}$ and for all $a_1,\ldots ,a_n$ in *N* and all formulas $\phi (x_1,\ldots ,x_n)$ of $L^d_A$ we have

$$\begin{aligned} \mathcal {M}\models \phi (a_1,\ldots ,a_n)\Rightarrow \mathcal {N}\models \phi (a_1,\ldots ,a_n).\end{aligned}$$

Respectively, we write $\mathcal {N}\preccurlyeq ^+_{L^d_A}\mathcal {M}$ if $\mathcal {N}\subseteq \mathcal {M}$ and for all $a_1,\ldots ,a_n$ in *N* and all formulas $\phi (x_1,\ldots ,x_n)$ of $L^d_A$ we have

$$\begin{aligned} \mathcal {N}\models \phi (a_1,\ldots ,a_n)\Rightarrow \mathcal {M}\models \phi (a_1,\ldots ,a_n).\end{aligned}$$

Similar definitions can be given for $L^{d,\omega }_A$ and $L^{d,\infty }_A$, and for fragments (i.e. subsets closed under subformulas) $\Gamma$ thereof.

The Compactness Theorem implies that every infinite model $\mathcal {N}$ has arbitrarily large $\mathcal {M}$ such that $\mathcal {N}\preccurlyeq ^+_{L^d_A}\mathcal {M}$ (Corollary 9).

### Theorem 12

(Downward Löwenheim-Skolem-Tarski Theorem) Suppose $\kappa \ge \aleph _0$, $A\subseteq 2^\omega$, $\mathcal {M}$ is a model for a vocabulary of cardinality $\le \kappa$, and $X\subseteq M$ such that $|X|\le \kappa$. Then there is $\mathcal {N}\preccurlyeq ^-_{L^d_A}\mathcal {M}$ (even $\mathcal {N}\preccurlyeq ^-_{\Gamma }\mathcal {M}$ for any fixed fragment $\Gamma$ of $L^{d,\infty }_A$ of size $\le \kappa$) such that $X\subseteq N$ and $|N|=\kappa$.

### Proof

We first expand $\mathcal {M}$ as follows: For every $L^d_A$-formula $\phi (R,\bar{z})$, where *R* is *n*-ary and $\bar{z}=z_0,\ldots ,z_{k-1}$, we make sure there is a predicate symbol $R^*$ of arity $k+n$ such that if $\mathcal {M}\models \exists R\phi (R,\bar{c})$, then $\mathcal {M}\models \phi (R^*(\bar{c},\cdot ),\bar{c})$. Likewise, we may assume the vocabulary of $\mathcal {M}$ has a Skolem function $f_\phi$ for each formula $\phi (x,\bar{z})$ such that if $\mathcal {M}\models \exists x\phi (x,\bar{c})$, then $\mathcal {M}\models \phi (f_\phi (\bar{c}),\bar{c})$. Let $\tau$ be the original vocabulary of $\mathcal {M}$ and $\tau ^*$ the vocabulary of the expansion, which we denote $\mathcal {M}^*$. For any formulas $\bar{\psi }$ in $L^d_A$ of the vocabulary $\tau ^*$ and $\bar{c}\in M^k$ such that (1) in Definition 5 holds, let $g(n,k,\bar{\psi }, \eta ,\bar{c})$ be the function which maps $n,k,{\bar{\psi }},\bar{c}$ and $\eta \in 2^n$ to $\langle b^n_0,\ldots ,b^n_{n-1}\rangle \in M^n$ such that $\mathcal {M}^*\models \Gamma ^{n,k}_{\bar{\psi },\eta \restriction n}(b^n_0,\ldots ,b^n_{n-1},a,\bar{c})$ for all $n<\omega$. Denoting for any cardinal $\theta$ the set of sets of hereditary cardinality $<\theta$ by $H_\theta$, let $\theta \ge (\kappa +2^\omega )^+$such that $M\subseteq H_\theta$, and $K\prec H_\theta$, such that $|K|=\kappa$ and $\{A,\kappa ,\tau ,\mathcal {M}^*, X,g\}\cup \kappa \cup \tau ^*\cup X\subseteq K$. Let $\mathcal {N}$ be the restriction of $\mathcal {M}^*$ to *K*, i.e. the universe *N* of $\mathcal {N}$ is $M\cap K$ and the constants, relations and functions of $\mathcal {M}^*$ are relativized to *N*.

We need to check that *N* is closed under the interpretations of function symbols of the vocabulary of $\mathcal {M}^*$. Let *f* be such a function symbol. Suppose *f* is *s*-ary and $\bar{c}\in N^s$. The sentence $\exists x(x\in M\wedge x=f^{\mathcal {M}^*}(\bar{c}))$ is true in $H_\theta$, hence true in *K*. Thus there is $b\in N (=M\cap K)$ such that $b=f^{\mathcal {M}^*}(\bar{c})$ is true in *K*. Therefore $f^{\mathcal {M}^*}(\bar{c})=b\in N$. We can conclude that $\mathcal {N}$ is a substructure of $\mathcal {M}^*$.

**Claim: ** If $\phi (\mathbf {x})$ is a $\tau$-formula in $L^d_A$ and $\mathbf {a}\in N$, then $\mathcal {M}^*\models \phi (\mathbf {a})\Rightarrow \mathcal {N}\models \phi (\mathbf {a})$.

We use induction on $\phi$. The claim follows from $\mathcal {N}\subseteq \mathcal {M}^*$ for atomic and negated atomic $\phi$. The claim is clearly preserved under conjunction and disjunction. It is also trivially preserved under universal quantifier, since $\mathcal {N}\subseteq \mathcal {M}^*$. The induction steps for both first and second order existential quantifiers are trivial because of the expansion we have performed on $\mathcal {M}^*$. We are left with the quantifier $Q_A$.

Suppose $\mathcal {M}^*$ satisfies (1) of Definition 5 with $\bar{c}\in N^k$. Thus (2) holds and we want to prove (2) with $\mathcal {M}^*$ replaced by $\mathcal {N}$. Note that (2) also holds in *K*. Suppose $\sigma \in 2^n$ is given. There is $\eta \in K\cap (2^\omega \setminus A)$ extending $\sigma$ such that *K* satisfies

$$\begin{aligned} \begin{array}{l} \text{ There } \text{ is }\, a\in M\, \text{ such } \text{ that } \text{ for } \text{ some } \text{ function }\, n\mapsto \langle b^n_0,\ldots ,b^n_{n-1}\rangle \\ \text{ from }\, \omega \, \text{ to }\, M^n\, \text{ we } \text{ have } \mathcal {M}^*\models \Gamma ^{n,k}_{\bar{\psi },\eta \restriction n}(b^n_0,\ldots ,b^n_{n-1},a,\bar{c}) \text{ for } \text{ all }\, n<\omega . \end{array} \end{aligned}$$

Thus there are $a\in N$ and a function $n\mapsto \langle b^n_0,\ldots ,b^n_{n-1}\rangle$ from $\omega$ to $N^n$ such that $\mathcal {M}^*\models \Gamma ^{n,k}_{\bar{\psi },\eta \restriction n}(b^n_0,\ldots ,b^n_{n-1},a,\bar{c})$ for all $n<\omega$. By the Induction Hypothesis, $\mathcal {N}\models \Gamma ^{n,k}_{\bar{\psi },\eta \restriction n}(b^n_0,\ldots ,b^n_{n-1},a,\bar{c})$ for all $n<\omega$ follows. $\square$

We conclude that every sentence of $L^d_A$ which has an infinite model has a countable model and an uncountable model.

The following examples show that Theorem 12 is in a sense optimal:

### Example 13

There is an uncountable model $\mathcal {M}$, namely $(\mathbb {P}(\omega ),a,\in )$, where *a* is the element $\omega$ of $\mathbb {P}(\omega )$, such that there is no countable model $\mathcal {N}$ with $\mathcal {N}\preccurlyeq ^+_{\Sigma ^1_1}\mathcal {M}$. There is a countable model $\mathcal {N}$, namely $(\omega ,<)$, such that there is no uncountable model $\mathcal {M}$ with $\mathcal {N}\preccurlyeq ^-_{\Sigma ^1_1}\mathcal {M}$.

## Proper extensions of $\Sigma ^1_1$ and $\Sigma ^1_{1,\delta }$

Our goal in this section is to show that for many $A\subseteq 2^\omega$ the logic $L^d_A$ properly extends $\Sigma ^1_1$ and $L^{d,\omega }_A$ properly extends $\Sigma ^1_{1,\delta }$. We have a spectrum of results to this effect but nothing as conclusive as being able to explicitly point out such a set *A*. There are obvious reasons for this. The logic $\Sigma ^1_1$ is very powerful and any “simple” $A\subseteq 2^\omega$ is likely to yield $L^d_A$ which is equivalent to $\Sigma ^1_1$ rather than properly extending it. This is even more true with $L^{d,\omega }_A$ and $\Sigma ^1_{1,\delta }$.

We first establish the basic existence of sets $A\subset 2^\omega$ with the desired properties. We shall then refine the result with further arguments.

### Theorem 14

There are sets $A\subseteq 2^\omega$ such that $2^\omega \setminus A$ is dense and $Q_A$ is not definable in $\Sigma ^1_1$, nor in $\Sigma ^1_{1,\delta }$, nor in $L_{\omega _1\omega _1}$.

### Proof

Let $A_\alpha$, $\alpha <2^\omega$, be disjoint dense subsets of $2^\omega$. For any $X\subseteq 2^\omega$, let

$$\begin{aligned}A_X=\bigcup _{\alpha \in X}A_\alpha .\end{aligned}$$

Note that if $X\ne Y$, then $A_X\setminus A_Y$ or $A_Y\setminus A_X$ is dense. Let $K_A$ and $K_B$ be as in Example 7 and $\mathcal {M}_A$ is as in Example 3. If $A\setminus B$ is dense, then $K_A\ne K_B$, as $\mathcal {M}_A\in K_B$ but $\mathcal {M}_A\notin K_A$. Thus the classes $K_{A_X}$, $X\subseteq 2^\omega$, are all different. For cardinality reasons there is $X\subseteq 2^\omega$ so that $K_{A_X}$ is not definable in $\Sigma ^1_{1,\delta }$, nor in $L_{\omega _1\omega _1}$. But $K_{A_X}$ is always definable in $L^d_{A_X}$. $\square$

The following result merely improves the previous result:

### Theorem 15

There is a countable $A_0\subseteq 2^\omega$ such that if $A_0\subseteq A\subseteq 2^\omega$ with $2^\omega \setminus A$ dense, then $Q_A$ is not $\Sigma ^1_1$-definable.

### Proof

Let us consider $\mathcal {M}_{B}$, where $B=2^\omega$ (defined in Example 3). Let $\mathcal {M}$ be resplendent (see [2]) such that $\mathcal {M}_B\prec \mathcal {M}$. Let $\mathcal {M}^+$ be an expansion of $\mathcal {M}$ such that every $\Sigma ^1_1$-sentence true in $\mathcal {M}^+$ has a witness in the vocabulary (countable). Let $\mathcal {N}^+\prec \mathcal {M}^+$ be countable. Let $A_0$ be the countable set $\Omega (\mathcal {N}^+)$. Suppose now $A_0\subseteq A\subseteq 2^\omega$, but $\psi _A$, as defined in Example 7, is definable by a $\Sigma ^1_1$-sentence $\phi$. Since $2^\omega \setminus A$ dense, $\mathcal {M}_B\models \psi _A$, whence $\mathcal {M}_B\models \phi$. Hence all the first order consequences of $\phi$ are true in $\mathcal {M}_B$. Since $\mathcal {M}$ is resplendent, $\mathcal {M}\models \phi$. Since $\phi$ has a witness in $\mathcal {M}^+$, $\mathcal {N}^+\models \phi$. Hence $\mathcal {N}^+\models \psi _A$ whence $\Omega (\mathcal {N}^+)\setminus A$ is dense. This is a contradiction, as $\Omega (\mathcal {N}^+)\setminus A=\emptyset$. $\square$

### Theorem 16

Let $\mathbf{P}$ be the poset of finite partial functions $(\omega _1+\omega _1)\times \omega \rightarrow 2$ i.e. the forcing for adding $\omega _1+\omega _1$ Cohen reals. Let *G* be $\mathbf{P}$-generic and $\eta _\alpha \in 2^\omega$, $\alpha <\omega _1+\omega _1$, the Cohen reals added by *G*. Let *A* be the set of $\eta$ such that $\eta =\eta _\alpha \text{(mod } \text{ finite) }$ for some $\alpha <\omega _1$. Then in *V*[*G*], $Q_A$ is not $\Sigma ^1_1$-definable.

### Proof

Let *B* be the set of $\eta$ such that $\eta =\eta _\alpha (\text{ mod } \text{ finite})$ for some $\alpha <\omega _1+\omega _1$. Then $\mathcal {M}_B\models \psi _A$. Suppose $\phi$ is a $\Sigma ^1_1$-sentence logically equivalent to $\psi _A$. Thus $\mathcal {M}_B\models \phi$. Let *f* be a bijection (in *V*) of $\omega _1+\omega _1$ onto $\omega _1$. The function *f* induces an complete embedding $\bar{f}$ of $\mathbf{P}$ into $\mathbf{P}$. The mapping $\bar{f}$ induces a mapping $\tau \mapsto \tau _{\bar{f}}$ between $\mathbf{P}$-terms. Let $\mathcal {N}$ be the image of $\mathcal {M}_B$ under this mapping. Now $\mathcal {N}\not \models \psi _A$. However, $\mathcal {N}\models \phi$, whence $\mathcal {N}\models \psi _A$, a contradiction. $\square$

### Theorem 17

Assume $A=2^\omega \setminus D$, where $D \subseteq 2^\omega$ is dense, $\omega<|D|< 2 ^ {\aleph _0}$ and there is an open set *U* such that $D\cap V$ is uncountable for every non-empty open $V\subseteq U$. Then the quantifier $Q_ A$ is not $\Sigma ^1_1$-definable.

### Proof

Recall that $\psi _A$ is a sentence of $L^d_A$ in the vocabulary $\tau _d$ saying that $\Omega (M)\setminus A$ is dense. Thus $\psi _A$ says $\Omega (M)\cap D$ is dense.

Let $\phi$ be a $\Sigma ^1_1$ sentence $\exists R\phi _0$ such that $\psi _A$ and $\phi$ are logically equivalent, contradicting our desired conclusion. Let $\langle D_ \alpha : \alpha < \omega _1\rangle$ be a sequence of disjoint countable dense subsets of $D \cap U$. Let $\mathcal {N}_ \alpha$ be a countable model representing the set $D_ \alpha$, whence it satisfies $\psi _A$, hence $\phi$, and there is an expansion $\mathcal {N}^*_ \alpha$ of $\mathcal {N}_ \alpha$ to a model of $\phi _0$. Let $\mathcal {N}=\langle N^*_ \alpha : \alpha < \omega _1 \rangle$. Let $\mathcal {B}=(H_\theta , \in , <)$, for a large enough cardinal $\theta$ and for a well-ordering < of $H_\theta$. We choose a countable elementary submodel $\mathcal {B}^*$ of $\mathcal {B}$ such that $\{\mathcal {N},A,\omega _1\}\subset \mathcal {B}^*$.

By Theorem IV.5.19 of [12] there is a sequence $\langle \mathcal {B} _ \alpha : \alpha < 2^{\omega }\rangle$ of countable elementary extensions of $\mathcal {B}^*$ such that for every $\alpha<\beta < 2^\omega$:

- $$\mathcal {B} _ \alpha $$ has standard $$ \omega $$.
- $\mathcal {B} _ \alpha$ has a (possibly non-standard) member $c_ \alpha$ of $(\omega _1)^{\mathcal {B}^* }$.
- If an element of ${}^{ \omega } 2$ is definable in both $\mathcal {B} _ \alpha$ and $\mathcal {B} _ {\beta }$, then it is in $\mathcal {B}^*$.

Let $\mathcal {N}_ \eta ^+$ be the $c_ \eta$’th member of the sequence $\langle \mathcal {N}^*_ \alpha : \alpha < \omega _1 \rangle$ as interpreted in $\mathcal {B} _ \eta$. So necessarily $\mathcal {N}_ \eta ^+$ is a model of $\phi _0$ and hence its reduct $\mathcal {N}_ \eta ^+\restriction \tau _d$ is a model of $\phi$, and further of $\psi _A$. We have continuum many models $\mathcal {N}_ \eta ^+\restriction \tau _d$ of $\psi _A$. However, we will now show that the number of $\eta$ for which the model $\mathcal {N}_ \eta ^+\restriction \tau _d$ satisfies $\psi _A$ is at most $|D|<2^\omega$, a contradiction. Suppose $\mathcal {N}_\eta ^+\restriction \tau _d\models \psi _A$. Then the subset of $2^\omega$ represented by $\mathcal {N}_\eta ^+=(\mathcal {N}^*_{c_\eta })^{\mathcal {B}_\eta }$, i.e. $(D_{c_\eta })^{\mathcal {B}_\eta }$, meets *D* in a dense set. Every element of $(D_{c_\eta })^{\mathcal {B}_\eta }$ is definable in $\mathcal {B} _ \eta$. By the disjointness clause (c) above we get the claimed contradiction.

We now finish the proof of Theorem 17: Suppose $\eta$ is such that $\mathcal {N}_\eta ^+\not \models \psi _A$. This is a contradiction because $\mathcal {N}_\eta ^+\models \phi$. $\square$

## No strongest extension

We show that there is no strongest extension among positive logics of first order logic, or $\Sigma ^1_1$, or $\Sigma ^1_{1,\delta }$, with the Compactness Theorem and the Downward Löwenheim-Skolem Theorem.

We consider sequences $\mathcal {A}=\langle A_\alpha :\alpha \le \omega _1\rangle$ such that each $A_\alpha$, $\alpha <\omega _1$, is a countable dense subset of $2^\omega$, $\alpha <\beta$ implies $A_\alpha \subset A_\beta$, $A_{\omega _1}=\bigcup _{\alpha <\omega _1}A_\alpha$, and the set $S=\{\alpha<\omega _1 : A_\alpha =\bigcup _{\beta <\alpha }A_\beta \}$ is stationary.

Let $\Theta _\mathrm{TL}$ be the first order sentence

$$\begin{aligned}&\exists x(R_3(x)\wedge \forall y(\lnot R_4(y)\vee R_2(x,y)))\\&\quad \wedge \forall x\forall y(\lnot R_0(x,y)\vee R_2(x,y))\\&\quad \wedge \forall x\forall y(\lnot R_1(x,y)\vee R_2(x,y))\\&\quad \wedge \forall x\forall y(\lnot R_2(x,y)\vee (R_4(x)\wedge R_4(y)))\\&\quad \wedge \forall x (\lnot R_4(x)\vee R_2(x,x))\\&\quad \wedge \forall x\forall y\forall z(\lnot R_2(x,y)\vee \lnot R_2(y,z)\vee R_2(x,z))\\&\quad \wedge \forall x\forall y\forall z(\lnot R_2(y,x)\vee \lnot R_2(z,x)\vee R_2(y,z)\vee R_2(z,y)). \end{aligned}$$

Intuitively, $\Theta _\mathrm{TL}$ says that $R_2$ is a tree-like partial order extending $R_0$ and $R_1$. For example, the model $\mathcal {M}_A$ of Example 3 always satisfies $\Theta _\mathrm{TL}$. If $\mathcal {M}\models \Theta _\mathrm{TL}$, then one element *a* of *M* can represent only one $\eta$, i.e.

5 $$\begin{aligned} \eta ,\eta '\in \Omega (\mathcal {M},a)\, \text{ implies }\, \eta =\eta '. \end{aligned}$$

### Definition 18

We define the Lindström quantifier $Q_\mathcal {A}$ as follows. Suppose $\mathcal {M}$ is a model and $\bar{c}\in M^k$. Then we define that $\mathcal {M}$ satisfies

6 $$\begin{aligned} (Q_\mathcal {A}x_0x_1)(\psi _0(x_0,x_1,\bar{c}),\psi _1(x_0,x_1,\bar{c}),\psi _2(x_0,x_1,\bar{c}),\psi _3(x_0,\bar{c}),\psi _4(x_0,\bar{c})) \end{aligned}$$

if and only if $\mathcal {M}_{\bar{\psi }}\models \Theta _\mathrm{TL}$ and $\Omega (\mathcal {M}_{\bar{\psi }})\cap A_{\omega _1}\in \mathcal {A}$, where $\mathcal {M}_{\bar{\psi }}$ is as in Definition 5 and $\Omega (\mathcal {M}_{\bar{\psi }})$ is as in Definition 4.

### Definition 19

We define $L^d_\mathcal {A}$ as the closure of first order logic under $\wedge ,\vee ,\exists$, $\forall ,\exists R$ and $Q_\mathcal {A}$. The fragment, where $Q_\mathcal {A}$ is applied to first order formulas $\bar{\psi }$ only is denoted $L^{d^-}_\mathcal {A}$. Similarly, $L^{d,\omega }_\mathcal {A}$, $L^{d,\infty }_\mathcal {A}$, $L^{d^-,\omega }_\mathcal {A}$, and $L^{d^-,\infty }_\mathcal {A}$.

### Theorem 20

(Łoś Lemma for $L^d_\mathcal {A}$) Suppose ${\mathcal {M}}_i$, $i\in I$, are models and *D* is an $\omega _1$-incomplete ultrafilter on a set *I*. Let ${\mathcal {M}}=\prod _{i\in I}{\mathcal {M}}_i/D$, $f_0,\ldots ,f_{n-1}\in \prod _{i\in I}M_i$ and $\phi (x_0,\ldots ,x_{n-1})$ in $L^d_\mathcal {A}$ (even in $L^{d,\infty }_\mathcal {A}$). Then

$$\begin{aligned} \{i\in I : {\mathcal {M}}_i\models \phi (f_0(i),\ldots ,f_{n-1}(i))\}\in D\Rightarrow {\mathcal {M}}\models \phi (f_0/D,\ldots ,f_{n-1}/D). \end{aligned}$$

### Proof

We follow the proof of Theorem 8. The only point that requires attention is the induction step for $Q_\mathcal {A}$. We assume

7 $$\begin{aligned} J=\{u\in I : \mathcal {M}_i\models Q_\mathcal {A}x_0x_1\psi _0(x_0,x_1,\bar{f}(i)) \ldots \psi _4(x_0,\bar{f}(i))\}\in D \end{aligned}$$

and demonstrate $M\models Q_\mathcal {A}x_0x_1\psi _0(x_0,x_1,\bar{f}/D) \ldots \psi _4(x_0,\bar{f}/D)$. As in the proof of Theorem 8, it can be shown that the set *B* of $\eta \in 2^\omega$ such that there is $a\in M$ such that for some $b^n_0,\ldots ,b^n_{n-1}$ in $\prod _iM_i/D$ we have $\mathcal {M}\models \Gamma ^{n,k}_{\bar{\psi },\eta \restriction n}(b^n_0,\ldots ,b^n_{n-1},a,\bar{f}/D)$ for all $n<\omega$, is the full set $2^\omega$. It follows that $2^\omega \cap A_{\omega _1}=A_{\omega _1}$ and hence that $2^\omega \cap A_{\omega _1}\in \mathcal {A}$, as claimed. $\square$

### Corollary 21

If $2^\omega \setminus A$ is dense, then $L^d_\mathcal {A}$ (even $L^{d,\infty }_\mathcal {A}$) satisfies the (full) Compactness Theorem.

### Proof

The ultrafilter we used in the proof of Corollary 9 was regular, hence $\omega _1$-incomplete. $\square$

We can prove the Downward Löwenheim-Skolem-Tarski Theorem for $L^{d^-}_\mathcal {A}$ only (see Proposition 25 and Theorem 27).

### Theorem 22

(Downward Löwenheim-Skolem-Tarski Theorem) Suppose $\mathcal {M}$ is a model for a countable vocabulary and $X\subseteq M$ is countable. Then there is $\mathcal {N}\preccurlyeq ^-_{L^{d^-}_\mathcal {A}}\mathcal {M}$ (even $\mathcal {N}\preccurlyeq ^-_{\Gamma }\mathcal {M}$ for any fixed countable fragment of $L^{d,\omega }_\mathcal {A}$) such that $X\subseteq N$ and $|N|\le \aleph _0$.

### Proof

We first expand $\mathcal {M}$ as follows: For every $L^{d^-}_\mathcal {A}$-formula $\phi (R,\bar{z})$, where *R* is *n*-ary and $\bar{z}=z_0,\ldots ,z_{k-1}$, there is a predicate symbol $R^*$ of arity $k+n$ such that if $\mathcal {M}\models \exists R\phi (R,\bar{c})$, then $\mathcal {M}\models \phi (R^*(\bar{c},\cdot ),\bar{c})$. Likewise, we may assume the vocabulary of $\mathcal {M}$ has a Skolem function $f_\phi$ for each formula $\phi (x,\bar{z})$ such that if $\mathcal {M}\models \exists \phi (x,\bar{c})$, then $\mathcal {M}\models \phi (f_\phi (\bar{c}),\bar{c})$. Let $\tau$ be the original vocabulary of $\mathcal {M}$ and $\tau ^*$ the vocabulary of the expansion, which we also denote $\mathcal {M}$. For any formulas $\bar{\psi }$ in $L^{d-}_\mathcal {A}$ of the vocabulary $\tau ^*$ let $g(n,k,\bar{\psi }, \eta )$ be the function which maps $n,k,{\bar{\psi }}$ and $\eta \in 2^n$ to $\Gamma ^{n,k}_{\bar{\psi },\eta }(y_0,\ldots ,y_n,x,\bar{z})$. Recall that $S=\{\alpha<\omega _1 : A_\alpha =\bigcup _{\beta <\alpha }A_\beta \}$ is stationary. Let $K\prec H_\theta$, where $\theta \ge (2^\omega )^+$ such that $M\subseteq H_\theta$, $|K|=\aleph _0$, $\{\mathcal {A},\omega _1,\tau ^*,\mathcal {M}, X,g\}\cup \omega _1\cup \tau \cup X\subseteq K$, and $\delta =K\cap \omega _1\in S$. Let $\mathcal {N}$ be the restriction of $\mathcal {M}$ to *K*, i.e. the universe *N* of $\mathcal {N}$ is $M\cap K$ and the constants, relations and functions of $\mathcal {M}$ are relativized to *N*.

As in the proof of Theorem 12, *N* is closed under the interpretations of function symbols of the vocabulary of $\mathcal {M}$.

**Claim: ** If $\phi (\mathbf {x})$ is a $\tau ^*$-formula in $L^{d^-}_\mathcal {A}$, then $\mathcal {M}\models \phi (\mathbf {c})\Rightarrow \mathcal {N}\models \phi (\mathbf {c})$.

We use induction on $\phi$. In light of the proof of Theorem 12, we only need to consider the quantifier $Q_\mathcal {A}$. Suppose $\mathcal {M}$ satisfies (6) with $\bar{c}\in N^k$. Thus $\Omega (\mathcal {M}_{\bar{\psi }})\cap A_{\omega _1}\in \mathcal {A}$. Hence

$$\begin{aligned} K\models ``\Omega (\mathcal {M}_{\bar{\psi }})\cap A_{\omega _1}\in \mathcal {A}''.\end{aligned}$$

Note that since $\delta \in S$, $K\cap A_{\omega _1}=A_\delta .$

**Case 1:** $\Omega (\mathcal {M}_{\bar{\psi }})\cap A_{\omega _1}=A_\alpha$ for some $\alpha <\omega _1$. Then $\alpha <\delta$ and

$$\begin{aligned} K\models ``\Omega (\mathcal {M}_{\bar{\psi }})\cap A_{\omega _1}=A_\alpha ''.\end{aligned}$$

We prove $\Omega (\mathcal {N}_{\bar{\psi }})\cap A_{\omega _1}=A_\alpha ,$ from which $\Omega (\mathcal {N}_{\bar{\psi }})\cap A_{\omega _1}\in \mathcal {A}$ follows.

Let first $\eta \in \Omega (\mathcal {N}_{\bar{\psi }})\cap A_{\omega _1}$. There are $a\in N$ and $b^n_0,\ldots ,b^n_{n-1}$ in *N* such that $\mathcal {N}\models \Gamma ^{n,k}_{\bar{\psi },\eta \restriction n}(b^n_0,\ldots ,b^n_{n-1},a,\bar{c})$ for all $n<\omega$. Since the formulas of $\bar{\psi }$ are first order, $\mathcal {M}\models \Gamma ^{n,k}_{\bar{\psi },\eta \restriction n}(b^n_0,\ldots ,b^n_{n-1},a,\bar{c})$ for all $n<\omega$. Hence $\eta \in \Omega (\mathcal {M}_{\bar{\psi }})\cap A_{\omega _1}=A_\alpha .$

For the converse, let $\eta \in A_\alpha$. Note that now $\eta \in K$. By the choice of $\alpha$, $\eta \in \Omega (\mathcal {M}_{\bar{\psi }})$. Hence there is $a\in M$ such that for some $b^n_0,\ldots ,b^n_{n-1}$ in *M* we have $\mathcal {M}\models \Gamma ^{n,k}_{\bar{\psi },\eta \restriction n}(b^n_0,\ldots ,b^n_{n-1},a,\bar{c})$ for all $n<\omega$. Such an *a* and such $b^n_0,\ldots ,b^n_{n-1}$ exist also in *K*, by elementarity, as $\eta \in K$. By Induction Hypothesis, $\mathcal {N}\models \Gamma ^{n,k}_{\bar{\psi },\eta \restriction n}(b^n_0,\ldots ,b^n_{n-1},a,\bar{c})$ for all $n<\omega$. Thus $\eta \in \Omega (\mathcal {N}_{\bar{\psi }})$.

**Case 2:** $\Omega (\mathcal {M}_{\bar{\psi }})\cap A_{\omega _1}=A_{\omega _1}$. Then $K\models ``\Omega (\mathcal {M}_{\bar{\psi }})\cap A_{\omega _1}=A_\delta ''.$ We prove $\Omega (\mathcal {N}_{\bar{\psi }})\cap A_{\omega _1}=A_\delta ,$ from which $\Omega (\mathcal {N}_{\bar{\psi }})\cap A_{\omega _1}\in \mathcal {A}$ follows.

Let first $\eta \in \Omega (\mathcal {N}_{\bar{\psi }})\cap A_{\omega _1}$. As in Case 1, $\eta \in \Omega (\mathcal {M}_{\bar{\psi }})$. Because we have (5), that is, $\eta$ is determined by an element of *N*, we may conclude $\eta \in K$. By $K\prec H_\theta$, $\eta \in (A_{\omega _1})^K$. Hence $\eta \in A_\delta$.

For the converse, let $\eta \in A_\delta$. Since $\delta \in S$, $A_\delta \subset K$, and hence $\eta \in K$. On the other hand, $\eta \in \Omega (\mathcal {M}_{\bar{\psi }})$, since $A_\delta \subset A_{\omega _1}=\Omega (\mathcal {M}_{\bar{\psi }})\cap A_{\omega _1}$. Now we can argue as in Case 1 to conclude $\eta \in \Omega (\mathcal {N}_{\bar{\psi }})$. $\square$

A consequence of Corollary 20 and Theorem 22 is that the positive logic $L^{d^-}_\mathcal {A}$ is an extension of $\Sigma ^1_1$ with both the Compactness Theorem and the Downward Löwenheim-Skolem Theorem. Similarly, $L^{d^-,\omega }_\mathcal {A}$ is such an extension of $\Sigma ^1_{1,\delta }$.

### Theorem 23

There are positive logics $L_1$ and $L_2$ such that

1. $L_1,L_2$ both (properly) extend $\Sigma ^1_1$.
2. $L_1,L_2$ both satisfy the Compactness Theorem and the Downward Löwenheim-Skolem Theorem.
3. There is no logic $L_3$ such that $L_1\le L_3$, $L_2\le L_3$, and $L_3$ satisfies the Downward Löwenheim-Skolem Theorem.

We can replace $\Sigma ^1_1$ by $\Sigma ^1_{1,\delta }$.

### Proof

Let $\mathcal {A}$ be as above but $A_\alpha =\bigcup _{\beta <\alpha }A_\beta$ for all limit $\alpha$. Let $S,S'\subseteq \omega _1$ be disjoint stationary sets. Note that the set of elements of *S* that are limits of elements of *S* is stationary, because it contains the intersection of *S* with the closed unbounded set of limits of elements of *S*. Similarly, the set of elements of $S'$ that are limits of elements of $S'$ is stationary. Let  and . Now both $\{\alpha \in S:A_\alpha =\bigcup _{\beta \in \alpha \cap S}A_\beta \}$ and $\{\alpha \in S':A_\alpha =\bigcup _{\beta \in \alpha \cap S'}A_\beta \}$ are stationary. Let

$$\begin{aligned} \psi _{\mathcal {A}}=(Q_{\mathcal {A}}x_0x_1)(R_0(x_0,x_1),R_1(x_0,x_1),R_2(x_0,x_1),R_3(x_0),R_4(x_0))\end{aligned}$$

and similarly $\psi _{\mathcal {A}'}$. Let $\phi$ be the sentence $\psi _{\mathcal {A}}\wedge \psi _{\mathcal {A}'}\wedge \Theta _{TL}$. This sentence has a model, namely $\mathcal {M}_{A_{\omega _1}}$. Suppose it has a countable model $\mathcal {N}$. Then $\Omega (\mathcal {N})\cap A_{\omega _1}\in \mathcal {A}\cap \mathcal {A}'$. Hence $\Omega (\mathcal {N})\cap A_{\omega _1}= A_{\omega _1}$. Since $\mathcal {N}\models \Theta _{TL}$, *N* must be uncountable, a contradiction. $\square$

### Corollary 24

No extension of $\Sigma _1^1$ is strongest with respect to the Compactness Theorem and the Downward Löwenheim-Skolem Theorem, among positive logics.

We shall now prove that Theorem 22 does not hold with $L^{d-}_\mathcal {A}$ replaced by $L^d_\mathcal {A}$.

### Proposition 25

Suppose $\mathcal {A}$ is as above. There is an uncountable model $\mathcal {M}$ for a countable vocabulary such that there is no countable $\mathcal {N}\preccurlyeq ^-_{L^{d}_\mathcal {A}}\mathcal {M}$.

### Proof

Let *M* be the union of ${2}^\omega$, ${2}^{<\omega }$ and ${2}^\omega \times \omega _1$. The relations of the structure $\mathcal {M}$ are

($$i=0,1$$).
2. $$R_2^{\mathcal {M}}=\{(a,b)\in (2^{<\omega })\times 2^\omega : a\triangleleft b\}$$.
3. $$R_3^{\mathcal {M}}=\{\emptyset \}.$$
4. $$R_4^{\mathcal {M}}={2}^\omega \cup {2}^{<\omega }.$$
5. $$R_5^{\mathcal {M}}=\{(a,(a,\alpha )): a\in 2^\omega , \alpha <\omega _1\}.$$
6. $$R_6^{\mathcal {M}}={2}^\omega .$$
7. $$R_7^{\mathcal {M}}={2}^{<\omega }.$$
8. $$Q_1^{\mathcal {M}}={2}^{\omega }\times \omega _1.$$
9. $$Q_2^{\mathcal {M}}=\{(a,\alpha )\in Q_1: (a\in A_0\wedge \alpha<\omega )\vee (a\in A_2\setminus A_1\wedge \alpha <\omega _1)\}.$$
10. $$Q_3^{\mathcal {M}}=\{(a,b) : \exists \alpha (a\in A_\alpha \wedge b\in A_{\omega _1}\setminus A_\alpha )\}.$$

Suppose $\mathcal {N}\preccurlyeq ^-_{L^{d}_\mathcal {A}}\mathcal {M}$ is countable. Let $\phi (x)$ be the existential second order formula

$$\begin{aligned} R_6(x)\wedge \exists F(F\, \text{ is } \text{ a } \text{ one-one } \text{ function } \text{ from }\, R_7\, \text{ onto }\, \{y:Q_2(x,y)\}). \end{aligned}$$

- $\mathcal {M}\models \phi (a)$ if and only if $a\in A_0^{\mathcal {M}}$.
- $\mathcal {N}\models \phi (a)$ if and only if $a\in A_0^{\mathcal {N}}\cup (A_2^{\mathcal {N}}\setminus A_1^{\mathcal {N}})$.

Thus

$$\begin{aligned} \mathcal {M}\models (Q_\mathcal {A}x_0x_1)(\psi _0(x_0,x_1,\bar{c}),\psi _1(x_0,x_1,\bar{c}),\psi _2(x_0,x_1,\bar{c}),\psi _3(x_0,\bar{c}),\psi _4(x_0,\bar{c}))\end{aligned}$$

but

$$\begin{aligned}\mathcal {N}\not \models (Q_\mathcal {A}x_0x_1)(\psi _0(x_0,x_1,\bar{c}),\psi _1(x_0,x_1,\bar{c}),\psi _2(x_0,x_1,\bar{c}),\psi _3(x_0,\bar{c}),\psi _4(x_0,\bar{c})) \end{aligned}$$

$$\square $$

Despite the negative result of Theorem 25, Theorem 22 still holds for the fragment of $L^d_\mathcal {A}$ obtained by dropping existential second order quantifiers.

### Definition 26

Let $L^{d0}_\mathcal {A}$ be defined as $L^d_\mathcal {A}$ (Definition 19) except that existential second order quantification is not allowed. Let $L^{d1}_\mathcal {A}$ be defined as the extension of $L^d_\mathcal {A}$ by adding negation to the logical operations.

Clearly, $L^{d0}_\mathcal {A}$ is a positive logic and it satisfies the Compactness Theorem because even $L^d_\mathcal {A}$ does. The logic $L^{d1}_\mathcal {A}$ is an abstract logic in the sense of [10]. Unlike our positive logics, it is closed under negation and also closed under substitution. Note that $L^{d0}_\mathcal {A}\le L^{d1}_\mathcal {A}$.

### Theorem 27

(Downward Löwenheim-Skolem-Tarski Theorem) Suppose $\mathcal {M}$ is a model for a countable vocabulary and $X\subseteq M$ is countable. Then there is $\mathcal {N}\preccurlyeq _{L^{d1}_\mathcal {A}}\mathcal {M}$ such that $X\subseteq N$ and $|N|\le \aleph _0$. In particular, $\mathcal {N}\preccurlyeq _{L^{d0}_\mathcal {A}}\mathcal {M}$.

### Proof

This is as in the proof of Theorem 22. We first expand $\mathcal {M}$ as follows: For every $L^{d1}_\mathcal {A}$-formula $\phi (\bar{z})$, where $\bar{z}=z_0,\ldots ,z_{k-1}$, there is a predicate symbol $R_\phi$ of arity *k* such that $\mathcal {M}\models \forall \bar{z}(\phi (\bar{z})\leftrightarrow R_\phi (\bar{z}))$. Let $\tau$ be the original vocabulary of $\mathcal {M}$ and $\tau ^*$ the vocabulary of the expansion. For any atomic formulas $\bar{\psi }$ of the vocabulary $\tau ^*$ let $g(n,k,\bar{\psi }, \eta )$ be the function which maps $n,k,{\bar{\psi }}$ and $\eta \in 2^n$ to $\Gamma ^{n,k}_{\bar{\psi },\eta }(y_0,\ldots ,y_n,x,\bar{z})$. Let $K\prec H_\theta$, where $\theta \ge (2^\omega )^+$ such that $M\subseteq H_\theta$, $|K|=\aleph _0$, $\{\mathcal {A},\omega _1,\tau ^*,\mathcal {M}, X,g\}\cup \omega _1\cup \tau \cup X\subseteq K$, and $\delta =K\cap \omega _1\in S$. Let $\mathcal {N}$ be the restriction of $\mathcal {M}$ to *K*, i.e. the universe *N* of $\mathcal {N}$ is $M\cap K$ and the constants, relations and functions of $\mathcal {M}$ are relativized to *N*.

As in the proof of Theorem 12, *N* is closed under the interpretations of function symbols of the vocabulary of $\mathcal {M}$.

**Claim: ** If $\phi (\mathbf {x})$ is a $\tau ^*$-formula in $L^{d1}_\mathcal {A}$ and $\mathbf {c}\in N$, then $\mathcal {N}\models \phi (\mathbf {c})\leftrightarrow R_\phi (\mathbf {c})$.

The proof of this claim is as in the proof of Theorem 22. Since $\mathcal {N}\subseteq \mathcal {M}$ in the vocabulary $\tau ^*$, the claim implies $\mathcal {N}\preccurlyeq _{L^{d1}_\mathcal {A}}\mathcal {M}$. $\square$

The logic $L^{d1}_\mathcal {A}$ is closed under negation and satisfies the Downward Löwenheim-Skolem Theorem. Thus it cannot satisfy the Compactness Theorem, although its sublogic $L^{d0}_\mathcal {A}$ does.

## Footnotes

1 The $(\kappa ,\lambda )$-Compactness Theorem says: Every theory of size $\le \kappa$, every subset of size $<\lambda$ of which has a model, has a model. Compactness Theorem means $(\omega ,\omega )$-Compactness Theorem.

2 The Downward Löwenheim-Skolem Theorem down to $\kappa$ says: Every sentence in a countable vocabulary, which has a model, has a model of size $\le \kappa$. “Down to $<\kappa$” means “has a model of size $<\kappa$”. The Downward Löwenheim-Skolem Theorem means the Löwenheim-Skolem Theorem down to $\aleph _0$.

3 By strongest extension we mean one which contains every other as a sublogic.

4 The Substitution Property for a abstract logic $L^*$ says that if $\phi$ is in $L^*$, *P* is an *n*-ary predicate symbol in the vocabulary of $\phi$ and $\psi (x_1,\ldots ,x_n)$ is a formula of $L^*$, then the result of substituting $\psi (t_1,\ldots ,t_n)$ to occurrences of $P(t_1,\ldots ,t_n)$ in $\phi$ is again in $L^*$. For details, see [6,  Def. 1.2.3].

5 An abstract logic (or “a generalized first order logic”), in the sense of [10] is a pair $L=(\Sigma ,T)$, where $\Sigma$ is an arbitrary set and *T* is a binary relation between members of $\Sigma$ on the one hand and structures on the other. Members of $\Sigma$ are called *L*-sentences. Classes of the form $\{\mathcal {M}:T(\phi ,\mathcal {M})\}$, where $\phi$ is an *L*-sentence, are called *L*-characterizable classes. Abstract logics are assumed to satisfy five axioms expressed in terms of *L*-characterizable classes. The axioms correspond to being closed under isomorphism, conjunction, negation, permutation of symbols, and “free” expansions.
